# Analog black holes and energy extraction by super-radiance from Bose Einstein condensates (BEC) with constant density

**DOI:** 10.1016/j.heliyon.2019.e02497

**Published:** 2019-09-30

**Authors:** Betül Demirkaya, Tekin Dereli, Kaan Güven

**Affiliations:** Department of Physics, Koç University, 34450 Sarıyer, İstanbul, Turkey

**Keywords:** Condensed matter physics, Vortex, Superradiance, Fluid dynamics, Bose-Einstein condensate

## Abstract

This paper investigates the acoustic superradiance of the density and phase fluctuations from the single vortex state of a Bose-Einstein condensate, by employing full time-domain and asymptotic frequency domain numerical calculations. The draining bathtub model of an incompressible barotropic fluid is adopted to describe the vortex. The propagation of the axisymmetric density and phase fluctuations in the condensate are governed by the massless scalar Klein-Gordon wave equation, which establishes the rotating black-hole analogy. Hence, the amplified scattering of these fluctuations from the vortex comprise the superradiance effect. A particular coordinate transformation is applied to reveal the event horizon and the ergosphere termwise in the metric and the respective asymptotic spectral solutions. A comparative analysis of the time domain and asymptotic frequency domain results are given for a range of rotational speed of the vortex and the frequency of the impinging fluctuations. The agreement at low rotational speeds of the vortex is shown to be very good, which starts to deteriorate at higher rotational speeds due to increasing constraint violations of the time-domain calculations. We further demonstrate an asymptotic upper bound for the superradiance as a function of vortex rotational speed, provided that the vortex remains stable.

## 1 Introduction

Analogies in physics enable us to observe a particular phenomenon with the same characteristic features in different systems pertaining to disparate mechanisms and space-time-energy scales. A particular example is the analogy between a cosmic black hole and the microscopic vortex state of a Bose-Einstein condensate, which casts the superradiance phenomenon of scalar waves from the black hole to the superradiance of acoustic waves from the liquid vortex. Superradiance allows for energy and angular momentum to be extracted from the vacuum, which coincides with the initial wave to have a reflection coefficient greater than one. Superradiance phenomena in rotating black holes first discovered by Zel'dovich in 1971 [1]. He showed that the amplifications occurs at the level of event horizon when a certain condition is met. In addition, the phenomena rely on the rotating black hole, described by the Kerr metric, which exhibits two key features; event horizon, described as null surface which acts as a one-way membrane and the ergoregion, stationary limit surface [2]. Since observing a cosmic scale superradiance is not a feasible option [3], we currently rely on analogous systems that can be realized at the laboratory scale. In this case a 2+1 space-time geometry Kerr black hole [4], [5], [6].

The analogy was initiated by Unruh's calculations [7], who showed the equivalence between the background solution of velocity perturbations on a perfect barotropic, irrotational Newtonian fluid and the Klein-Gordon field propagating in a 4-dimensional pseudo-Riemannian manifold, in which the speed of sound plays the role of speed of light. Because superradiance phenomenon occurs in the space-time background of rotating black holes, the analogy could be set for a rotating acoustic black-hole in a liquid [8], [9], [10], [11], [12]. Thus far, theoretical and computational investigation of the analogue superradiance have been reported for liquid systems [13], [14], relativistic fluids [15], shallow water systems [16] and optic systems [17], [18], [19], [20]. Within the context of BEC, superradiance stemming from the coherent light matter interaction [21], [22], as an analogue to the Dicke effect [23] and the amplification of matter waves as a manifestation of Raman superradiant scattering were also reported theoretically [24]. In addition, various physical phenomena such as black hole lasers have been adapted to analogue black hole systems [25], [26].

The experimental studies for that purpose emerged only within the last few years. Experimental realizations of horizons were reported in water channels [27], atomic Bose-Einstein condensates (BECs) [28]. Recently, rotational superradiant scattering in a water vortex flow is reported [29], [30]. Acoustic black hole in a needle-shaped BEC of 87Rb is realized and recently spontaneous Hawking radiation, stimulated by quantum vacuum fluctuations, emanating from an analogue black hole in an atomic Bose-Einstein condensate is reported [31], [32], [33].

In the perspective of the existing literature, different studies provide theoretical models to study the superradiance in the time domain and in the frequency domain. The methodology for the time domain solution is described for the Kerr black holes [34] and charged black holes [35] and modified for the draining-tub model of an acoustic black hole in irrotational, barotropic and incompressible fluid systems [36], [37]. While in the frequency domain, the superradiance is mainly analyzed at the asymptotic with an appropriate coordinate transformation [13], [14], [38]. The present work aims to contribute in two aspects: Providing a consolidating study of the temporal and spatial features of the scattering from a BEC vortex with constant background density and characterization of the superradiance as a function of the rotational speed of the vortex. We primarily adopt the draining bathtub model (DBT) introduced by Visser [11], to describe the acoustic black-hole in BEC vortex flow. The time domain solutions are obtained by solving the Klein-Gordon equation for the propagation of acoustic waves, whereas the spectral analysis of the superradiance is conducted by asymptotic solutions of the waves at the event horizon and the spatial infinity. The time-domain solutions are obtained by implementing the numerical techniques described mainly in Refs. [34], [37], [39]. Complementary to the cited works above, our study demonstrates very good agreement between the full time-domain and asymptotic frequency domain solutions and reveal novel spectral features within the DBT model. In particular, an asymptotic upper bound for the maximum reflection coefficient with respect to rotational speed of the vortex is obtained. The paper is organized as follows: Section 2 describes briefly the BEC system and gives a theoretical formulation leading to the main (Klein-Gordon) equation. Section 2.2 and 3 are devoted to the implementation and computation of the time-domain solutions. Section 3.1 presents the asymptotic solutions in the frequency domain. The last section discusses the main results and concludes the paper.

## 2 Model

We begin by a brief description of the Bose-Einstein condensate as the physical system of interest. A quantum system of *N* interacting bosons in which most of the bosons occupy the same single particle quantum state, the system can be described by a Hamiltonian of the form;

(1) $$H=\int dx{\overset{ˆ}{\Psi }}^{†}(t,x)\left[-\frac{\hbar^{2}}{2m}\nabla^{2}+V_{ext}(x)\right]\overset{ˆ}{\Psi }(t,x)+\frac{1}{2}\int dxdx^{\prime }{\overset{ˆ}{\Psi }}^{†}(t,x){\overset{ˆ}{\Psi }}^{†}(t,x^{\prime })V(x-x^{\prime })\overset{ˆ}{\Psi }(t,x^{\prime })\overset{ˆ}{\Psi }(t,x).$$

Here $V_{ext}$ is an external potential, $V(x-x^{\prime })$ is the interatomic two-body potential, *m* is the mass of the bosons and ${\overset{ˆ}{\Psi }}^{†}(t,x)$ is the boson field operator which includes the classical contribution ψ(t,x) plus excitations $\overset{ˆ}{\varphi }$, where $\psi (t,x)\equiv \langle {\overset{ˆ}{\Psi }}^{†}(t,x)\rangle$ known as the wave function of the Bose-Einstein condensate.

In the non relativistic limit, most of the atoms occupy the ground state and the classical wave function, ψ(t,x) describes the system. The interatomic interaction is taken as $V(x-x^{\prime })=U_{0}\delta (x-x_{0})$, $U_{0}=4a\pi \hbar^{2}/m$, where the constant *a* is the scattering length constant. Closed-form equation for weakly interacting bosons, with the potential defined above leads to the time dependent Gross-Pitaevskii (GP) equation:

(2) $$i\hbar \frac{\partial \psi }{\partial t}=\left(-\frac{\hbar^{2}}{2m}\nabla^{2}+V_{ext}+U_{0}{\left|\psi \right|}^{2}\right)\psi (r,t).$$

Here in hydrodynamic form the wave function can be written in terms of its magnitude and phase:

(3) $$\psi (r,t)=\sqrt{\rho }e^{iS}.$$

Then, the density of particles is given by $\rho (t,r)={\left|\psi (t,r)\right|}^{2}$ and the background fluid velocity is defined as $\overset{\to }{\upsilon }=(\hbar /m)\nabla S$. A general review on BEC formulation can be found in [40], [41].

The Draining Bathtub Model introduced by Visser et al. [11] describes the single vortex state of the BEC, where the fluid velocity has tangential and radial components,

(4) $$\overset{\to }{\upsilon }=\upsilon_{\overset{ˆ}{\phi }}+\upsilon_{\overset{ˆ}{r}}=\frac{-A}{r}\overset{ˆ}{r}+\frac{B}{r}\overset{ˆ}{\phi }$$

with *A* and *B* are constants to be determined.

The density and phase fluctuations are introduced respectively as $\rho =\rho_{0}+\rho_{1}$ and $S=S_{0}+S_{1}$, where $\rho_{0}$ and $S_{0}$ define the background. For small fluctuations, the GP equation can be linearized perturbatively, leading to the propagation equations of the fluctuations as follows:

(5) $$\frac{\partial \rho_{1}}{\partial t}+\frac{\hbar }{m}\nabla \cdot (\rho_{0}\nabla S_{1})+\nabla \cdot (\rho_{1}\upsilon )=0,$$

(6) $$\partial_{t}S_{1}=-\upsilon \cdot \nabla S_{1}-\frac{U_{0}}{\hbar }\rho_{1}+\frac{\hbar }{2m}D_{2}\rho_{1},$$

where $D_{2}\rho_{1}$ is given by

(7) $$D_{2}\rho_{1}=\frac{1}{2\sqrt{\rho_{0}}}\nabla^{2}\frac{\rho_{1}}{\sqrt{\rho_{0}}}-\frac{\rho_{1}}{2\rho_{0}^{3/2}}\nabla^{2}\sqrt{\rho_{0}}.$$

In the equation above Eq. (6), the pressure term $U_{0}\rho_{1}$ is of the order $U_{0}\rho /R$ while the quantum pressure term $\frac{\hbar^{2}}{2m}D_{2}\rho_{1}$ is of the order $\hbar^{2}/mR^{3}$, where *R* is the spatial scale [42]. This implies that for

(8) $$R>>\frac{\hbar }{\sqrt{2mU_{0}\rho }}\equiv \xi$$

the last term on the right-hand side of Eq. (6), is negligibly small. The right side of equation 8 thus introduces a characteristic length scale, known as the healing length. In the hydrodynamic approximation, equations (5) and (6) are combined to yield

(9) $$\frac{\partial }{\partial t}\left[\frac{\rho_{0}}{c^{2}}\left(\frac{\partial S_{1}}{\partial t}+\overset{\to }{\upsilon }\cdot \nabla S_{1}\right]\right)-\nabla \cdot \left(\rho_{0}\nabla S_{1}\right)+\nabla \cdot \left[\frac{\rho_{0}}{c^{2}}\left(\frac{\partial S_{1}}{\partial t}+\overset{\to }{\upsilon }\cdot \nabla S_{1}\right)\right]=0$$

where the speed of sound is defined by $c=\sqrt{\rho U_{0}/m}$. For a constant background density profile, the speed of sound, *c*, is constant. Equation (9) then becomes equivalent to the massless Klein Gordon equation, describing the propagation of linear density and phase fluctuations in the axially symmetric 2+1 space-time. The metric associated with this space-time will be

(10) $$ds^{2}=\frac{\rho_{0}}{c}\left[-(c^{2}-\frac{A^{2}+B^{2}}{r^{2}})dt^{2}+\frac{2A}{r}dtdr-2Bdtd\phi +dr^{2}+r^{2}d\phi^{2}+dz^{2}\right].$$

### 2.1 Coordinate transformations

The coordinate transformation given below is particularly useful to minimize the number of off-diagonal components of the metric, leaving only one.

(11) $$dt=dt^{⁎}-gdr\;d\phi =d\phi^{⁎}-hdr\;r=r^{⁎}\;z=z^{⁎},$$

where $h=-(AB)/(r(A^{2}-c^{2}r^{2}))$ and $g=-(Ar)/(A^{2}-c^{2}r^{2})$. We drop the *-superscript in the following part of the formulation. The line equation takes the form

(12) $$ds^{2}=\frac{\rho_{0}}{c}[-\left(1-\frac{A^{2}+B^{2}}{c^{2}r^{2}}\right)dt^{2}+{\left(1-\frac{A^{2}}{c^{2}r^{2}}\right)}^{-1}dr^{2}-\frac{2Bd\phi dt}{c}+r^{2}d\phi^{2}+dz^{2}].$$

The event horizon and the ergosphere can be identified termwise in the metric now. For a stationary and axisymmetric spacetime metric, the radius of the ergosphere is given by the vanishing of $g_{00}$ and the coordinate singularity of the metric signifies the event horizon. From Eq. (12), they read as

(13) $$r_{event}=A/c,\;r_{ergo}={\left(A^{2}+B^{2}\right)}^{1/2}/c>r_{event}.$$

We assign the event horizon to *a* making, A=ac and $B=\Omega a^{2}$ and scale radial coordinate by r/a and time coordinate by tc/a.

### 2.2 Numerical solution in the time domain

In order to solve the Eq. (9), first we write the line element in the form;

(14) $$ds^{2}=-\alpha^{2}dt^{2}+\gamma_{ij}(dx^{i}+\beta^{i}dt)(dx^{j}+\beta^{j}dt),$$

where α=c, $\gamma_{ij}=diag(1,r^{2},1)$ and $\beta^{i}=(A/r,-B/r^{2},0)$. From this point, we change the notation for $S_{1}(x_{i},t)$ to Ψ to simplify the equations below, i.e. $\Psi =S_{1}(r,t)$. Equation (6) with the hydrodynamic approximation applied gives the density fluctuation as $\rho_{1}=-\frac{\hbar }{U_{0}}\partial_{t}\Psi -\frac{\hbar }{U_{0}}\upsilon \cdot \nabla \Psi$. Substituting velocity from Eq. (4) and scaling by the background density and the healing length Eq. (8) gives

(15) $$\frac{\rho_{1}}{(\sqrt{2}\xi )\rho_{0}}=-\frac{1}{c}\frac{\partial \Psi }{\partial t}+\frac{A}{cr}\frac{\partial \Psi }{\partial r}-\frac{B}{cr^{2}}\frac{\partial \Psi }{\partial \phi }.$$

For numerical calculations two conjugate fields are introduced as follows

(16) $$\Phi =\frac{\partial \Psi }{\partial x^{i}}\;\Pi =-\frac{1}{\alpha }\left(\frac{\partial \Psi }{\partial t}-\beta^{i}\Phi_{i}\right),$$

where $\Psi =\psi_{1}(t,r)e^{im\phi }e^{ikz}$, $\Pi =\pi_{1}(t,r)e^{im\phi }e^{ikz}$ and $\Phi =\phi_{1}(t,r)e^{im\phi }e^{ikz}$ and (m,k) are the axial and azimuthal wave numbers, respectively [34], [43]. In this work, in accordance with the BEC vortex stability conditions, we consider the azimuthal wave numbers of m=0 and 1 only [44], [45]. Using the conjugate fields, we obtain a hyperbolic system of three coupled first order partial differential equations.

(17) $$\partial_{t}\pi_{1}+c\partial_{r}\phi_{1}-\frac{A}{r}\partial_{r}\pi_{1}=-imB\pi_{1}/r^{2}+c(k^{2}+m^{2}/r^{2})\psi_{1}-c\phi_{1}/r$$

(18) $$\partial_{t}\psi_{1}-\frac{A}{r}\partial_{r}\psi_{1}=-imB\psi_{1}/r^{2}-c\pi_{1}$$

(19) $$\partial_{t}\phi_{1}+c\partial_{r}\pi_{1}-\frac{A}{r}\partial_{r}\phi_{1}=2imB\psi_{1}/r^{3}-(A+imB)\phi_{1}/r^{2}.$$

## 3 Results & Discussion

We first describe briefly the implementation numerical black-hole excision adopted from Refs. [46] and [37], to solve Eqs. (17)-(19). In this technique, a numerical boundary is placed beyond the event horizon (an “apparent horizon”) which excises its interior from the computational domain, thereby removing the singularity. Since no information can leave the interior of the black hole, the excision should have no effect on the physics outside. This translates to the numerical implementation as a constrained evolution in which the constraint violations occurring beyond the event horizon has to be monitored and kept below a tolerance. Consequently, the time-domain calculations are limited by a time scale until the constraint violations start to interfere with the physical computation domain beyond a tolerance. This time scale decreases with increasing rotational speed of the vortex and thus affects adversely the global settings of the time-domain calculations.

This main section is organized as follows: We first calculate the time evolution of the perturbations of the velocity potential by solving the equation set (17)-(19) and check the consistency of the numerical result at event horizon and the outer boundary. Second, the energy of the perturbations is calculated showing the superradiant and non-superradiant cases based on the ranges of the model parameters Ω and *ω*.

From the fields Π,Φ and *ψ*, we construct outgoing and incoming fields along the null ray

(20) $$u^{+}\propto \Pi +\Phi \;u^{-}\propto \Pi -\Phi .$$

At large distances purely outgoing wave is implemented such that $u^{-}=0$, $\pi_{1}=\phi_{1}$. We terminate the numerical simulation before the fluctuations reach to the outer boundary so that it has no effect in the obtained results. For calculating the scattering amplitude the outgoing wave is recorded at a specified location before the outer boundary. For the numerical boundary beyond the event horizon, free-end boundary conditions are applied, such that waves are free to propagate without any constraints. We remind that the relevant condition to be monitored is not this boundary but the constraint conditions evaluated at the excision horizon, in this case the event horizon. The positioning of the numerical boundary beyond the event horizon is subject to two criteria: It must be far from the r=0 singularity to reduce the amplification of the constraint violations beyond the event horizon and also far from the actual event horizon so that the constraint violations cannot propagate to the physical domain in a short time to interfere with the actual wave.

The computational spatial (radial) and time domain are set as 0.2<r/a<150,0<tc/a<150, with discretization steps of Δr=0.05, Δt=0.05, respectively.

(21) $$\psi_{1}(0,r)=Nexp\left[-{\left(r-r_{0}+ct\right)}^{2}/b^{2}-i\omega (r-r_{0}+ct)/c\right].$$

The incident wave is chosen as an axisymmetric imploding Gaussian pulse modulated by a monochromatic wave, centered at $r_{0}=50a$ with a width of b=10a and azimuthal wavenumber k=0 [43]. Here, *c* is the propagation speed of sound in the condensate and *a* is the location of the event horizon. Both parameters are scaled to unity and the amplitude *N* is calculated by the normalization of the incident wave on the radial domain.

We note that the location of the incident wave should be chosen numerically far enough so that the scattering outcome is independent from the location of the incident wave. The angular speed of the vortex is Ω. In the present calculations, we consider values of Ω=1.4c/a up to Ω=4.2c/a. The frequency of the incident wave is ω=Ω/2.

**Figure 1:**
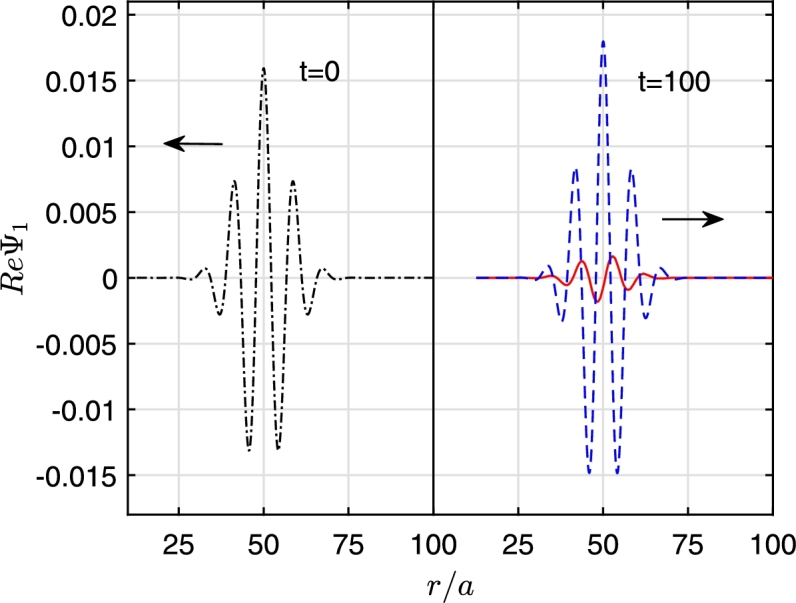
Incident wave at *t* = 0 (left) and the reflected wave at *t* = 100*a*/*c* for the superradiance case, amplified (dashed blue line) and non superradiant case (red line). The parameters used are *r*_0_ = 50*a* and *b* = 10*a* with *ω* = 0.7*c*/*a*, Ω = 1.4*c*/*a*.

Fig. 1 shows the snapshots of the initial cylindrical Gaussian wavepacket at t=0 and t=100a/c for both superradiant (m=1) and non-superradiant (m=0) cases. The perturbation for the non-superradiant case is close to zero while in the superradiant case it gets amplified. We monitor the constraint value *C*, from the definition of Φ, Eq. (16)

(22) $$C=\left|\partial_{r}\psi_{1}-\phi_{1}\right|.$$

**Figure 2:**
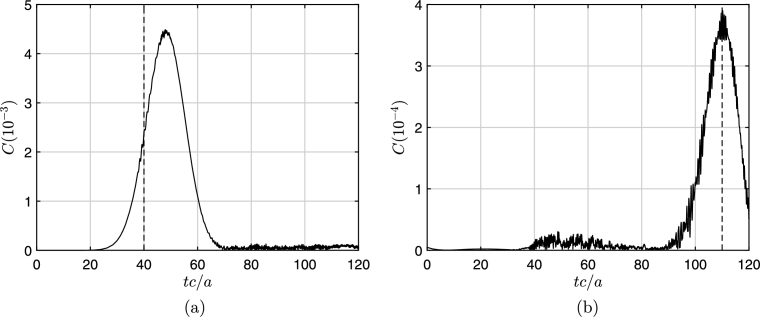
Constraint violations at event horizon (a) and outer boundary (*r* = 70*a*) (b) for superradiance (*m* = 1). The parameters used are in Fig. 1. Dotted lines signify the time frames (*tc*/*a* = 40,110), when the wave reaches the horizon and the outer boundary.

To analyze the reflection, we put a monitor at r/a=70 and record the outgoing wave at this location. Fig. 2 shows the constraint violations at the event horizon (left panels) and at the recording monitor's position (r/a=70) (right panels) for the superradiant case (m=1). Evidently, the violations are large when the wave passes from the respective location. For 40<tc/a<70 note how the constraint violations at the outer boundary are also enhanced while the wave interacts with the event horizon. However, we observe that the overall magnitude of the constraint violations are about an order of magnitude higher at the event horizon compared to the outgoing wave monitor location (r/a=70).

**Figure 3:**
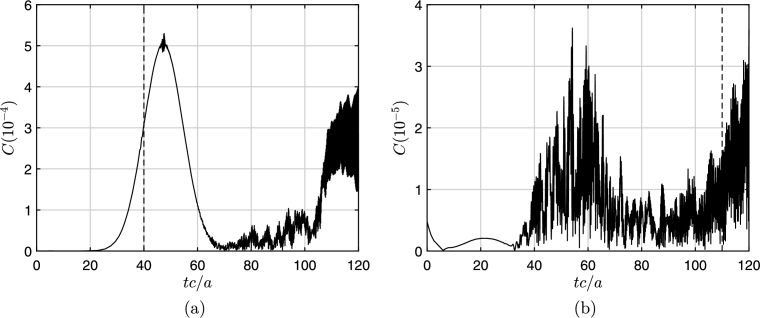
Constraint violations at event horizon (a) and outer boundary (*r* = 70*a*) (b) for non-superradiance (*m* = 0). The parameters used are in Fig. 1.

**Figure 4:**
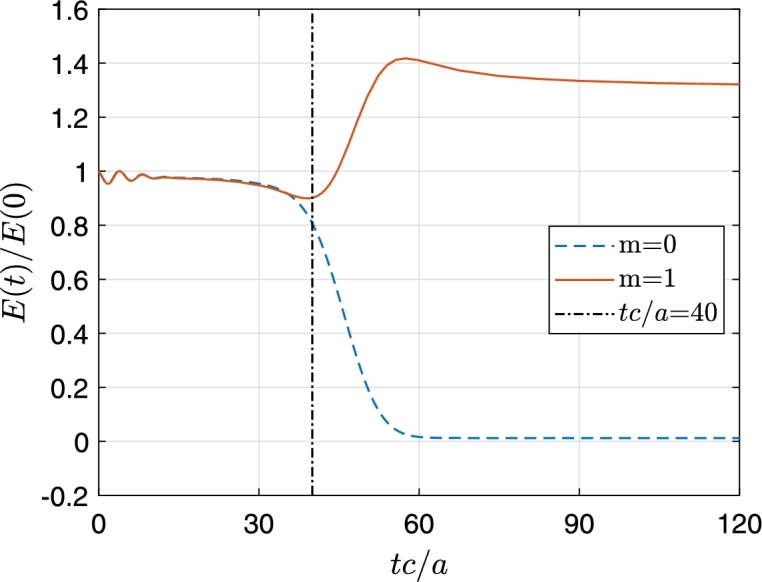
Time evolution of the energy gain of the wave packet, superradiant *m* = 1 case and non-superradiant *m* = 0 case. The parameters used are in Fig. 1.

Similarly, Fig. 3 shows the constraint violations for the non-radiant case (m=0). Fig. 3(a) exemplifies nicely the numerical problem associated with the excision technique: After the physical wave interacted with the event horizon (30<tc/a<70) the instabilities induced at the numerical boundary beyond the event horizon start to propagate out and reach to the event horizon around tc/a=100. Note that by this time the physical wave has reached almost to the outer boundary and is not affected by these instabilities. If the simulation had been allowed to continue further, these amplified instabilities would propagate further and render the results obsolete. The constraint violations depend on the rotational speed of the vortex (Ω) and the relative center frequency of the impinging wavepacket (ω/Ω). In general, high values of Ω and ω/Ω increases the overall magnitude of the constraint violation at the event horizon. Also the energy associated with the superfluid flow, energy of wave packet, is given as

(23) $$E(t)=\int \partial^{3}r\frac{1}{2}M\rho {\overset{\to }{\upsilon }}^{2}=(\rho \hbar^{2}/2M)\int_{0}^{2\pi }d\phi \int_{0}^{H}dz\int_{1}^{r_{max}}{\left(\nabla \psi_{1}\right)}^{2}rdr,$$

which is calculated, normalized by the energy of the initial wave and plotted in Fig. 4 for the non-radiant (dashed blue curve) and superradiant (solid red curve) cases respectively. The incident wave arrives to the event horizon approximately at t=40a/c.

**Figure 5:**
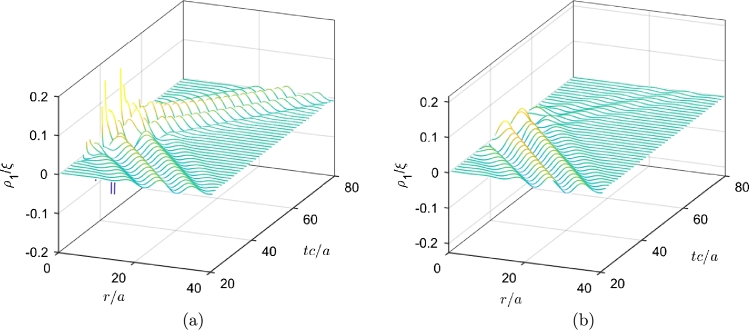
Density fluctuations, *ρ*_1_ in *r*-*t* plane for superradiance case, *m* = 1 (a) and non-superradiance case *m* = 0 (b).

The time evolution of the density fluctuations associated with the acoustic wave propagation are plotted in Fig. 5 for superradiant and non-radiant cases which shows the detailed view of the propagation of fluctuations near the event-horizon. Sudden increase in the density fluctuations for the superradiance case, stays inside the event horizon, r=1a.

**Figure 6:**
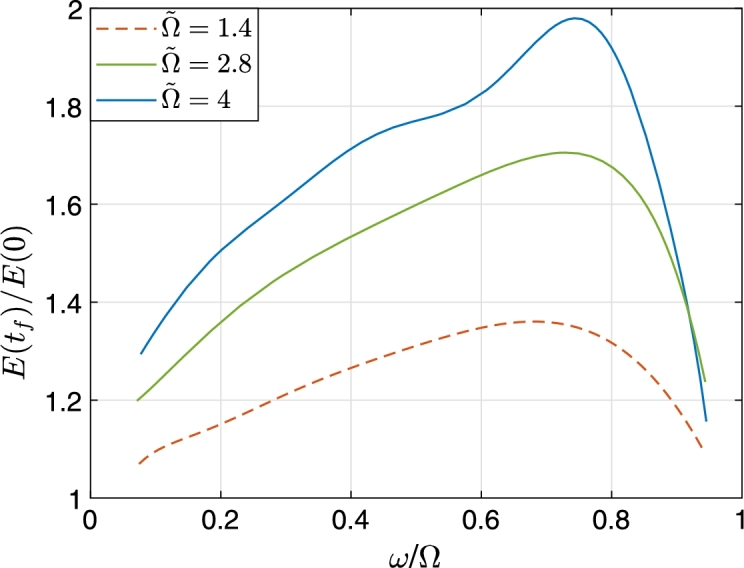
The energy calculated according to Eq. (23) normalized to its initial value *E*(*t* = 0) as a function of *ω*/Ω where 0 < *ω* < Ω_*i*_. The parameters used in the calculations are $\Omega_{i}=\overset{˜}{\Omega }c/a=1.4c/a$, 2.8*c*/*a*, 4*c*/*a*, *r*_0_ = 50*a*, *b* = 10*a*. Energy values recorded when the outgoing wave reached the monitor boundary at *r*/*a* = 50.

The amplification of energy as a function of the relative center frequency of the wave ω/Ω is plotted in Fig. 6, for values Ω=1.4c/a,2.8c/a,4c/a. The amplification gradually increases with a maximum typically in the range 0.6<ω/Ω<0.8 beyond which it decreases rapidly. The amplification curve shifts up with increasing Ω.

### 3.1 Numerical model in the frequency domain

In this section we analyze the Klein Gordon equation (Eq. (9)) in the frequency domain. Using separation of variables, the formal solution of the KG is expressed as

(24) $$\psi =e^{-i\omega t}e^{im\phi }e^{ikz}P(r),$$

where *k* and *m* are the axial and azimuthal wave numbers, respectively. To avoid polydromy problems [37], that is to make *ψ* single valued, *m* should be taken as an integer and *k* a real number defined by the boundary conditions along the *z* axis.

By inserting (24) into (9), we obtain a second order ODE for the radial part:

(25) $$\frac{d^{2}P}{dr^{2}}+\left(\frac{A^{2}+r^{2}c^{2}+2iA(Bm-r^{2}\omega )}{r(r^{2}c^{2}-A^{2})}\right)\frac{dP}{dr}+\left(\frac{2iABm-B^{2}m^{2}+c^{2}m^{2}r^{2}+2Bm\omega r^{2}-r^{4}\omega^{2}+c^{2}k^{2}r^{4}}{r^{2}(r^{2}c^{2}-A^{2})}\right)P=0.$$

We substitute $P=R(r)H(r_{⁎})$ with a Regge-Wheeler tortoise coordinate, $r_{⁎}$, which will map $r\in [r_{H},\infty ]$ to $r_{⁎}\in [-\infty ,+\infty ]$:

(26) $$r_{⁎}=\int \frac{r^{2}}{r^{2}-A^{2}/c^{2}}dr.$$

Introducing $r_{⁎}$ into Eq. (25) yields the final form of the equations for R(r) and $H(r_{⁎})$

(27) $$\frac{dR(r)}{dr}+\frac{A(2i(Bm-r^{2}\omega^{2})-A)+r^{2}c^{2}}{2r(r^{2}c^{2}-A^{2})}=0,$$

(28) $$\frac{d^{2}H(r_{⁎})}{d{r_{⁎}}^{2}}+\left(\frac{\omega^{2}}{c^{2}}-V(r)\right)H(r_{⁎})=0,$$

where

(29) $$V=k^{2}(1-\frac{A^{2}}{r^{2}c^{2}})-\frac{5A^{4}}{4c^{4}r^{6}}-\frac{A^{2}\left(m^{2}-3/2\right)+B^{2}m^{2}}{c^{2}r^{4}}-\frac{1}{4r^{2}c^{2}}\left(c^{2}-4m^{2}c^{2}-8B\omega \right).$$

Near the event horizon and at the far field (r→+∞), the asymptotic solutions are given by the harmonic functions,

(30) $$H(r_{⁎})=e^{\frac{i\omega_{+}r_{⁎}}{c}}+{Re}^{\frac{-i\omega_{+}r_{⁎}}{c}},r^{⁎}\overset{}{\to }+\infty$$

(31) $$H(r_{⁎})=Te^{\frac{-i(\omega -\Omega m)r_{⁎}}{c}},r^{⁎}\overset{}{\to }-\infty$$

where $\omega_{+}^{2}=\omega^{2}-k^{2}c^{2}$ and $B=\Omega A^{2}/c^{2}$ and R(T) are the amplitudes of the reflected (transmitted) waves, respectively. Here, in to achieve scattering states $\omega_{+}$ should be positive.

The equality of the Wronskian of these solution at asymptotics gives

(32) $$1-{\left|R\right|}^{2}=\left(\frac{\omega -m\Omega }{\omega_{+}}\right)\left|T^{2}\right|$$

It shows that when the superresonance condition, ω<mΩ, is satisfied, reflection coefficient has a magnitude larger than unity [47], [48]. Eq. (32) reveals the superradiance condition clearly (i.e. ω<mΩ) and gives the full spectral behavior of the reflection coefficient. Thus, we can obtain the reflection coefficient through the Fourier components of the asymptotic far field solution, which is obtained through Eq. (26) and Eq. (28).

**Figure 7:**
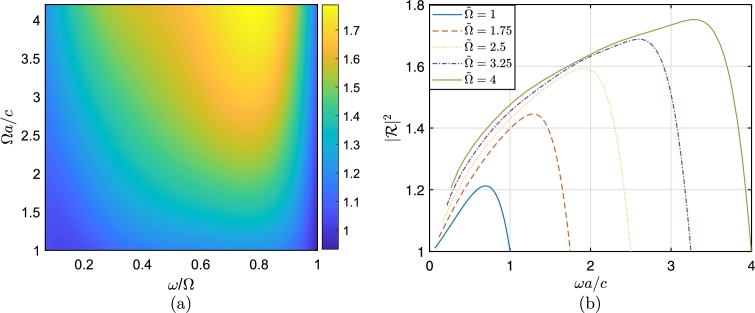
a) Reflection coefficient values between 1 < Ω*a*/*c* < 4.2 and Ω/15 < *ω* < Ω. b) Reflection coefficients as a function of *ω*, calculated in the range 0 < *ω* < *m*Ω_*i*_. Parameters are *m* = 1, and $\overset{˜}{\Omega }=\Omega_{i}a/c$ = 1, 1.75, 2.5, 3.25, 4.

Fig. 7(a) shows the variation of the reflection coefficient in the Ω-ω/Ω plane and 7(b) shows expanded slices at different Ω values (the horizontal axis spans multiple ranges). These calculations employ the model parameters (m,a,c,k,A,B) with the same values used in the time-domain calculations of section 3.

**Figure 8:**
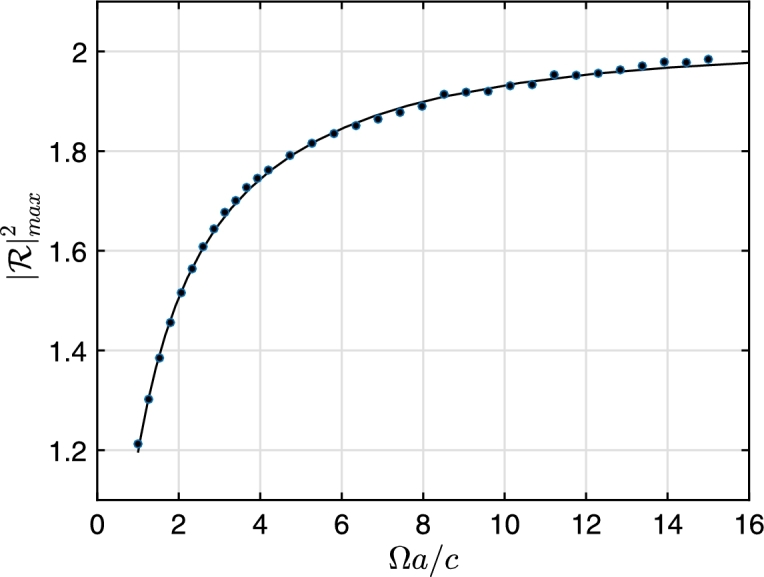
Maximum reflection coefficient behavior with respect to Ω between 1 < Ω*a*/*c* < 16.

**Figure 9:**
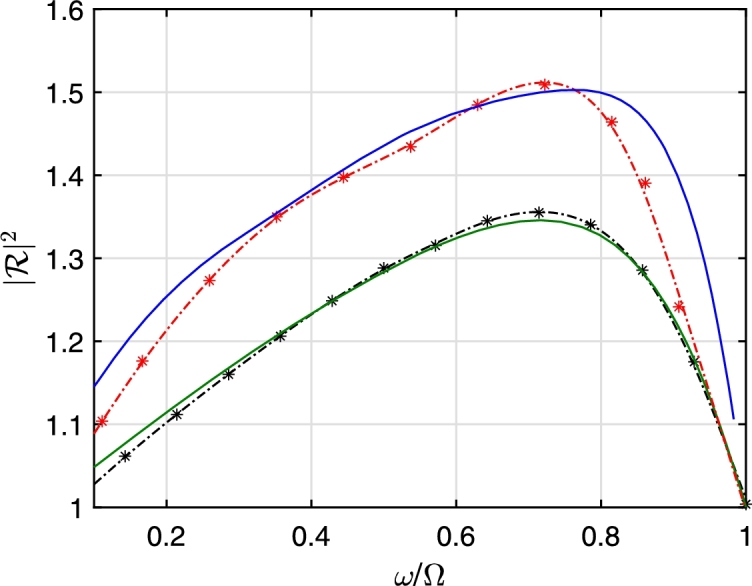
Dependence of the reflection coefficient to the frequency of the incident acoustic perturbation, scaled by the vortex frequency. Upper (lower) pair of curves are for Ω*a*/*c* = 2.8(=1.4). Solid curves are obtained from direct time-domain calculations. Dash-dotted lines are obtained from asymptotic frequency domain calculations.

Fig. 8 shows the maximum value of the reflection coefficient as a function of rotational speed of the vortex. Interestingly, the frequency domain calculations predict an upper bound for the maximum superradiance, which might be connected to entropy bound as stated in [49]. We stress however that at large rotational speeds, the stability of the vortex and hence this outcome becomes arguable. Fig. 9 the compares reflection coefficient calculated in the time-domain (section 3) and frequency domain (section 3.1), respectively for different values of Ω. As noted before, the time-domain calculations are susceptible to large values of Ω due to numerical instabilities, whereas the frequency domain is immune. The comparison justifies this observation: The agreement between the time-domain and frequency domain is very good at Ωa/c=1.4 and in the intermediate range of ω/Ω values.

## 4 Conclusion

In this work, we investigated the amplified scattering of acoustic waves propagating in a BEC, from a vortex state with a constant background density, by obtaining both time-domain and asymptotic frequency domain solutions numerically. Time-domain study amounts for solving the Klein-Gordon equation which governs the radial propagation of sound waves in the presence of vortex, in analogy to scalar field propagation in the curved space-time of a black-hole. It is worth to note that the classical (macroscopic) wave function of the BEC represents the classical space-time of General Relativity only when probed at long-enough wavelengths such that it behaves purely hydrodynamically. The frequency domain calculations are performed at the radial part of the solution transformed to the (-infinity, + infinity) range, and by introducing Fourier components of the asymptotic incoming and outgoing fields. The amplitudes of these fields yield the transmission and reflection coefficients at each frequency component.

The major outcomes of the study are as follows: The comparison of full time-domain solutions and asymptotic solutions in the frequency domain show that their agreement is good near the characteristic rotational speed of the vortex Ω. As Ω increases, the constraint equations introduced by the excision technique in the time-domain calculations become increasingly violated, rendering the results with large error margins. The frequency domain asymptotic solutions do not suffer from numerical instabilities and the scattering coefficient can be calculated for arbitrarily large values of Ω. This formulation predicts an upper bound of ${\left|R\right|}_{max}^{2}\approx 2$ against the rotational speed of the vortex, although the stability of the vortex at high rotational speeds is not taken into account.

As a final note, the methods presented in this work are suitable to employ non-constant background density profiles. Since the sound propagation speed will also vary, it is possible to explore novel features of the superradiance phenomenon from the BEC vortex beyond the constant-density approximation. This will be pursued in subsequent studies.

## Declarations

### Author contribution statement

Betül Demirkaya; Tekin Dereli; Kaan Güven: Conceived and designed the analysis; Analyzed and interpreted the data; Contributed analysis tools or data; Wrote the paper.

### Funding statement

Betül Demirkaya was supported by TUBITAK-BIDEB 2211 National Scholarship Program for PhD Students.

### Competing interest statement

The authors declare no conflict of interest.

### Additional information

No additional information is available for this paper.
